# Further perceptions of probability: Accurate, stepwise updating is contingent on prior information about the task and the response mode

**DOI:** 10.3758/s13423-024-02604-2

**Published:** 2024-11-14

**Authors:** Mattias Forsgren, Peter Juslin, Ronald van den Berg

**Affiliations:** 1 Department of Psychology, Uppsala University, P. O. Box 1225, 751 42 Uppsala, Sweden; 2 Department of Psychology, Stockholm University, Stockholm, Sweden

**Keywords:** Probability learning, Probability estimation, Hypothesis testing, Associative learning

## Abstract

To adapt to an uncertain world, humans must learn event probabilities. These probabilities may be stationary, such as that of rolling a 6 on a die, or changing over time, like the probability of rainfall over the year. Research on how people estimate and track changing probabilities has recently reopened an old epistemological issue. A small, mostly recent literature finds that people accurately track the probability and change their estimates only occasionally, resulting in staircase-shaped response patterns. This has been taken as evidence that people entertain beliefs about unknown, distal states of the world, which are tested against observations to produce discrete shifts between hypotheses. That idea stands in contrast to the claim that people learn by continuously updating associations between observed events. The purpose of this article is to investigate the generality and robustness of the accurate, staircase-shaped pattern. In two experiments, we find that the response pattern is contingent on the response mode and prior information about the generative process. Participants exist on continua of accuracy and staircase-ness and we only reproduce previous results when changing estimates is effortful and prior information is provided—the specific conditions of previous experiments. We conclude that explaining this solely through either hypotheses or associations is untenable. A complete theory of probability estimation requires the interaction of three components: (i) online tracking of observed data, (ii) beliefs about the unobserved “generative process,” and (iii) a response updating process. Participants’ overt estimates depend on how the specific task conditions jointly determine all three.

**Supplementary Information:**

The online version contains supplementary material available at 10.3758/s13423-024-02604-2.

## Introduction

**Fig. 1:**
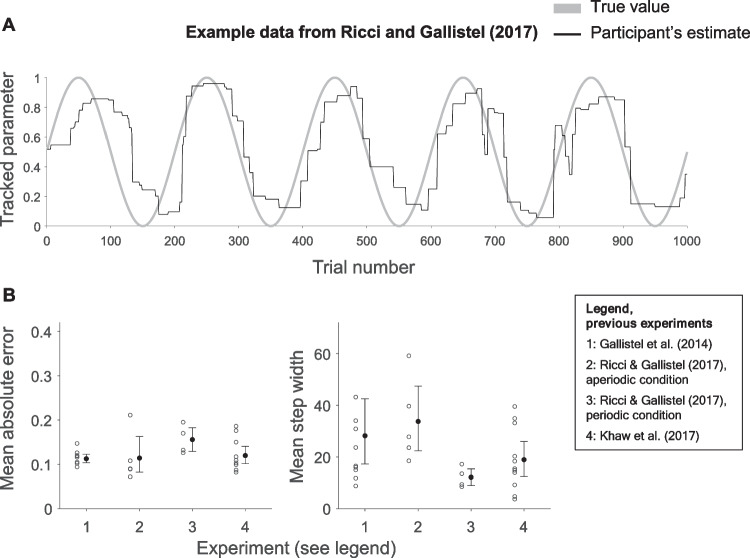
Examples from previous experiments. **A**: participant in Ricci & Gallistel (2017) who exhibits a staircase shaped response curve, with long periods without an update. The participant is tracking a Bernoulli parameter that changes according to a sinusoidal function. **B**: Mean absolute error and mean step widths for previous experiments. Dots indicate group means, whiskers indicate 95% bootstrapped confidence intervals based on 1,000,000 resamples. Circles indicate individual participants

A small, mostly recent literature has presented participants with a task where they track a latent, nonstationary Bernoulli parameter (Gallistel et al., 2014; Khaw et al., 2017; Ricci & Gallistel, 2017; Robinson, 1964, “Bernoulli paradigm”). Participants observe consecutive draws with replacement from the Bernoulli distribution and provide an estimate after each draw. Here, one consistently finds “stepwise” response behaviour: participants do not change their estimate for many trials, and when they do, they adjust by large or small steps (Fig. 1A). Participants are also often quite accurate, closely tracking the true latent probability. These accurate, “staircase shaped” response curves have been presented as a ubiquitous and robust phenomenon deriving from participants’ mental shifts between discrete hypotheses^1^—thus tying them to a long-standing debate between cognitivist and associationist frameworks (see Fodor, 1983).

For example, Ricci & Gallistel (2017) conclude that “we find that the step-hold pattern is seen in every subject... even when the characteristics of the stochastic process to which subjects are exposed discourages such a strategy” (p. 1482). Gallistel et al. (2014) suggest that “trial-by-trial updating models cannot explain the statistics of the step-like pattern of estimates seen in all subjects” (p. 99). Similarly, Khaw et al. (2017) report in the abstract that “subjects adjust in discrete jumps rather than after each new piece of information” (p. 88). These claims are surprising given the prominent role played by trial-by-trial updating models in related neuropsychological paradigms which do not find stepwise updating (e.g., McGuire et al., 2014; Nassar et al., 2010; Norton et al., 2019)—there, even demographics who update particularly infrequently (Bruckner et al., 2020; Nassar et al., 2021) update more frequently than the average participant in the Bernoulli paradigm literature.

Here, we will show that the accurate and stepwise response curves are in fact not ubiquitous even in the Bernoulli paradigm. Rather, participants exist on continua of more or less accurate responses and more or less stepwise updating and can be moved along these continua by adjusting design choices in the experiment task. We argue that this invites a theory that allows a role for trial-by-trial tracking of the observed events (e.g., associations), beliefs about the task (hypotheses), as well as an adaptable response process. Rather than either/or, people may simultaneously be intuitive statisticians (Peterson & Beach, 1967) and intuitive scientists (Szollosi & Newell, 2020) whose overt responses correspond only imperfectly to their covert representations.

We concentrate on two quantitative measures to capture the important properties of data of the sort presented in Fig. 1. One measure concerns the estimation error, calculated as the average absolute distance between the participant’s response and the true probability (a low error implies high accuracy). The other measure concerns the step widths in the response curves, calculated as the number of trials between changes to the estimate.^2^ As can be seen in Fig. 1B, the data from the periodic condition in Ricci & Gallistel (2017), for example, have a mean absolute error of 0.156 (95% bootstrapped confidence interval [0.129, 0.183], *N* = 4) and a mean step width of 12.2 trials (CI [8.98, 15.4], *N* = 4).

**Fig. 2:**
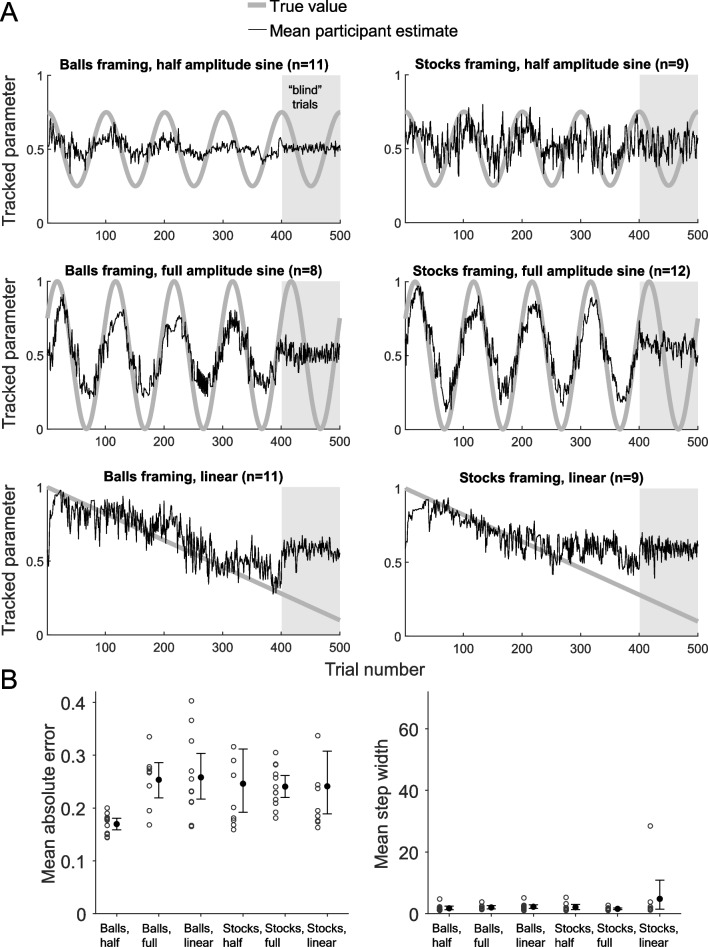
Results from an unpublished pilot study. **A**: Six groups of participants provided trial-by-trial estimates of the value of a non-stationary Bernoulli parameter based on observed outcomes from the Bernoulli process. Conditions differed in how the parameter changed over time (thick grey curves) and the cover story of what the Bernoulli process described (blue or green colour ball being drawn; stock market index going up or down). The last 100 trials were “blind”: the outcomes of the Bernoulli process were hidden to reveal if participants had inferred the underlying pattern. **B**: Means and 95% bootstrapped confidence intervals by condition for two summary statistics. Circles indicate individual participants. One outlier each in the Stocks, half and Stocks, linear condition with a mean absolute error of 0.460 and 0.459, respectively, is not displayed to preserve legibility

In a pilot study (unpublished), we tested 60 participants in a conceptual replication of the Bernoulli paradigm. Our version (Fig. 2) manipulated the functional form of the generative function and the cover story of the task (blue or green ball drawn vs. the stock market going up or down) between subjects. We also added a “blind phase” at the end of the experiment, where the outcomes were obscured and participants had to extrapolate from what they had learned. We had expected to find stepwise patterns similar to those in Fig. 1A. Surprisingly, however, accuracy was poorer than in other Bernoulli paradigm studies, there was little evidence of staircase-shaped response curves, and participants seemed unable to track the parameter in the blind phase (i.e., no signs of extrapolation based on an explicit hypothesis; see Fig. 2). This initial attempt made us suspect that stepwise updating may not be as robust a phenomenon as had been assumed.

This prompted us to further investigate its robustness through the two experiments reported below. An immediate limitation of Gallistel et al. (2014), Khaw et al. (2017), and Robinson (1964) is that the Bernoulli parameter itself changed in a staircase-shaped pattern in those studies. Hence, the discreteness seen in the response patterns might simply have reflected the discreteness in the stimulus patterns, rather than being a property of the cognitive process. Ricci & Gallistel (2017) addressed this confound by including one condition where the parameter changed periodically according to a sine function and one where it changed aperiodically.

In Experiment 1 we aimed for a direct replication of the periodic condition in Ricci & Gallistel (2017), but again we were unable to replicate the high accuracy and ubiquitous staircase curves. In Experiment 2, we scrutinized the remaining difference between our and previous experiments: explicitly telling the participants that the probability distribution was nonstationary.^3^ We also investigated the effect of the amount of effort required to select a new response, because we had noticed that this required more effort in the paradigm of Gallistel et al. (2014), Khaw et al. (2017), Ricci & Gallistel (2017), and Robinson (1964; i.e., the studies that found mostly stepwise updating) compared with our pilot and the paradigms of McGuire et al. (2014) and Nassar et al., (2010; i.e., the studies that found very little stepwise updating).

To preview our results, we find that response curves are noticeably affected by minor changes to the amount of effort required to change a response and instructions about the nonstationarity of the tracked parameter. Although some participants produce staircase-shaped response curves, this is not the general case. We take this as evidence that accurate, stepwise updating is not a ubiquitous and robust phenomenon but one that occurs more often in specific circumstances. In addition, we replicate previous anecdotal reports (Ricci & Gallistel, 2017) that participants can extract and report some description of the generative function. These results suggest to us that a complete theory of the cognitive process should include the ability to (i) track the association between observed events online, (ii) hold beliefs about the unobserved generative process, (iii) regulate when and how often the overt estimate is updated to correspond to the covert, internal estimate (see Forsgren et al., 2023), and adapt (i), (ii), and (iii) depending on available information and task interface.

## General methods

The experiment task was to repeatedly estimate a continuously changing Bernoulli parameter—the proportion of targets in a box—based on draws with replacement. The latent Bernoulli parameter changed over trials according to a sinusoidal function. Here, we describe the methods for the two main experiments. Methods, materials, and data for the pilot study and both experiments are available at https://osf.io/zhv2r/.

### General design and materials

**Fig. 3:**
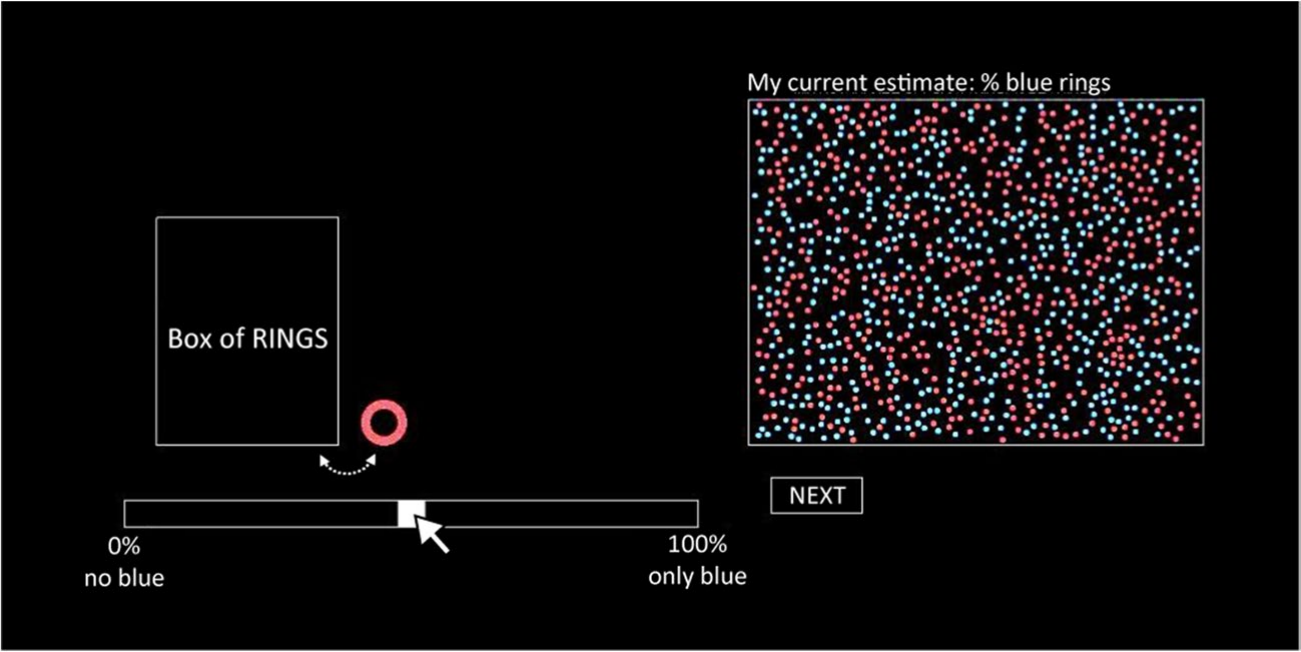
Experimental paradigm. Screenshot of our replication of the visual design of the experiments by Gallistel et al. (2014), Khaw et al. (2017), and Ricci & Gallistel (2017). All text translated from Swedish to English and slightly enlarged for readability

We replicated the interface described in Gallistel et al. (2014) and Ricci & Gallistel (2017).^4^ It consisted of a box labelled “Box of RINGS﻿”, a bar with a slider, and a rectangle filled with red and blue dots, which visualised the current slider value (Fig. 3). At the beginning of each trial, a single ring would be drawn from the box and remain visible for the duration of the trial. The task was to estimate the proportion of blue rings^5^ in the box using a slider on a bar labelled “0% = no blue” and “100% = only blue” on the left and right end, respectively. Adjusting the slider caused the proportion of red and blue dots in the rectangle labelled “My current estimate: % blue rings” to change to reflect the new proportion indicated by the slider position. This was intended as a visual aid to help the participants to “see” their currently chosen estimate. After locking in their response (see Design sections below for details on how), the trial ended by the ring being returned to the box. The animation of returning and then drawing a ring imposed a minimum trial time of about 1.1 seconds, depending on frame rate latency. Importantly, participants could choose to repeat their last estimate by locking in their response without adjusting the slider. The next trial commenced immediately after responding, starting with a new ring being drawn. The experiment consisted of 2000 trials with an optional break halfway through. The task was performed using the mouse. The mouse cursor was never repositioned automatically but remained where the participant positioned it.

Previous designs have varied in three details which we comment on here. Firstly, Gallistel et al. (2014) also included two buttons labelled “I think the box has changed” and “I take that back,” which participants could use to report declarative perceptions of there having been a discrete change in the latent probability distribution. We followed Ricci & Gallistel (2017), who argued that the buttons make little sense under continuously changing generative functions (where the box is changed every trial), in excluding them. Secondly, we removed the possibility of clicking left or right of the slider to make it jump a set distance, just like Khaw et al. (2017). This feature decreased response effort for responding with certain estimates while keeping them high for the rest. Removing it allowed unequivocal manipulation of the amount of effort required to update (see Experiment 2). Our participants could thus only move the slider via the mouse cursor (see Design sections below for details). Lastly, like Ricci & Gallistel (2017), we used the written instructions from Gallistel et al. (2014) but excluded the passages relating to reporting that the box had changed (since those buttons had been removed). The instructions were translated to Swedish by the first author (see https://osf.io/zhv2r/). All participants gave their informed consent to participate.

### Procedure

Participants read an informed consent form and the instructions for the task and confirmed to the experimenter that they had no questions. Importantly, they were not given any information regarding whether the content of the box would, could, or could not change throughout the course of the experiment. We manipulated availability of such prior information in Experiment 2. The participants then carried out the task (see Materials) for 1000 trials, before they were prompted to take a break. When they felt ready, participants commenced the final session of 1000 trials.^6^

After finishing the experiment, the participants filled out a questionnaire with questions concerning their beliefs about the generative function, self-assessed statistics proficiency, age, gender, and education. Finally, they were asked to draw a graph of what they thought the probability of drawing a blue ring had been as a function of trial count. We will return to these graphs in the General Discussion.^7^

### Analysis

Because of the nonnormal nature of our dependent variables (accuracy and step width), we used a (nonparametric) permutation approach adapted to analysis of variance (ANOVA) models (Anderson, 2001) for statistical analysis. We employed an unrestricted sampling algorithm (see Gonzalez & Manly, 1998; Howell, 2009; Manly, 2018) with 1,000,000 resamples but note here that other algorithms should produce comparable results (Gonzalez & Manly, 1998). The algorithm begins by calculating the *F* values for the main and interaction effects on the original data. Next, the data are shuffled 1,000,000 times, randomly redistributing the values among the cells of the factorial design. For each of the shuffled datasets, the *F* values are computed. This process generates an empirical null distribution of *F* values for each main effect and interaction. Finally, the *p* value for an effect is calculated as the proportion of *F* values in the null distribution that equal or exceed the *F* value observed in the data. For more detailed information, please refer to the code.

## Experiment 1

Experiment 1 was an attempt to exactly replicate the periodic condition in Ricci & Gallistel (2017). They used a sine function with a period of 200 trials to create a smoothly changing, periodic Bernoulli parameter that participants were to track. In addition to that condition, our experiment also included a condition in which the period of the sine wave was increased to 500. We expected this slower-changing parameter to be easier to track and thereby rule out that any pattern was an artefact of excessive difficulty.

### Method

#### Participants

Thirty-one participants (mean age = 25.9 years, *SD* = 7.07) were recruited from university campuses in Uppsala.^8^ The sample thus mainly consisted of students. To ensure naivety, participants were screened to confirm that none had participated in the pilot study. One participant completed Experiment 1 despite having done so; therefore, these data were excluded. Twenty-one valid participants identified as women and nine as men. Participants were rewarded with course credits or gift vouchers to a major Swedish cinema chain (SF bio) worth approximately US$29.^9^

#### Design

The experiment had two between-subjects conditions. For the first group, the generative function was a sinusoidal with a period of 200 trials (Short period), just like in the periodic condition in Ricci & Gallistel (2017). The only change for the second group was that the period was 500 trials (Long period).

Both groups provided their responses as in Gallistel et al (2014), Ricci & Gallistel (2017) and Khaw et al. (2017): by dragging the slider with the mouse cursor by pressing and holding the left mouse button. When they had dragged the slider to the desired position, they clicked a button labelled “Next” to lock in their response. The slider would remain in position between trials, why they could repeat their previous estimate by clicking “Next” without moving the slider.

#### Materials

The task was replicated from the code used in Ricci & Gallistel (2017). See General Methods.

#### Procedure

The procedure was as specified under General Methods.

### Results

**Fig. 4:**
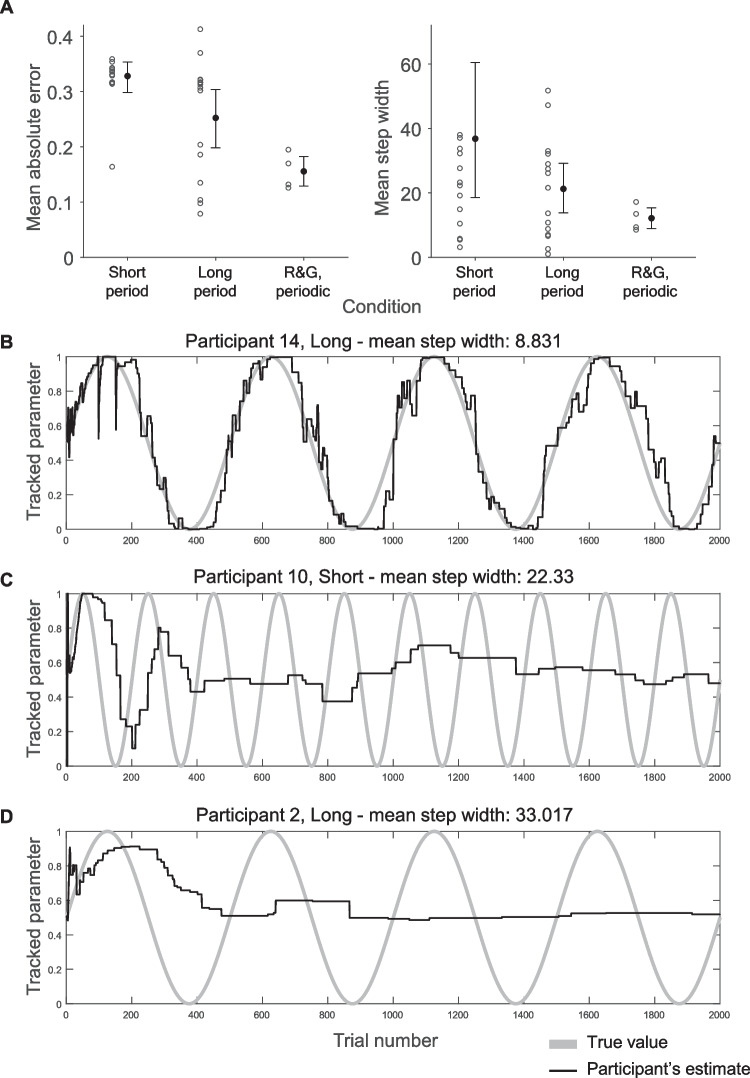
Results from Experiment 1. **A**: Mean absolute error and mean step width by condition and the periodic condition in Ricci & Gallistel (2017) – “R&G, periodic”. Whiskers indicate 95% bootstrapped confidence intervals, circles individual participants. One outlier each in the Short and Long condition with mean absolute error of 0.446 and 0.413, respectively, is not displayed to preserve legibility. Two outliers in the Short period condition with mean step widths of 136 and 143 are also off-scale. **B**, **C** & **D**: Data from the three participants whose mean step width was closest to the 25th, 50th, and 75th percentile, respectively. Note how participants in panel C and D have a high accuracy/low step width at the start of the experiment which then decreases/increases

We do not replicate the high accuracy (Short period: *mean* = 0.328, *SD* = 0.056; Long period: 0.252, 0.108; see Fig. 4A) from Ricci & Gallistel (2017), Gallistel et al. (2014), and Khaw et al. (2017): the upper limits of the bootstrapped confidence intervals of their data never exceed 0.20 (Fig. 1B), which is the lowest limit of the confidence intervals here (Fig. 4A). The bootstrapped confidence intervals for mean step-widths (Short period: 18.582, 60.472; Long period: 13.818, 29.185) overlap with most previous studies (Fig. 1B). Further, individual level data shows examples of response curves without the characteristic staircase shape (e.g. Fig. 4B). Altogether, the qualitative character of the response curves is different here than in previous experiments, with much poorer accuracy. Participants improve when there is a Long period (*p* = 0.023). There was no evidence, however, that step width is affected by Period length (*p* = 0.25). This indicates that increasing the period length indeed constituted a manipulation of task difficulty.

In the Supplement we report a decomposition of participants’ estimation errors into three sources: (i) insufficient number of updates; (ii) estimates lagging behind the changing parameter, and (iii) other. Not surprisingly, an insufficient number of updates accounts for more error when the latent parameter is changing more quickly (Short period: 32.5%, Long period: 12.4%) while the contribution from lag is more equal (10.5% vs. 14.4%), However, in both conditions, the largest proportion of error is attributable to other sources (57.0% vs. 73.2%).

### Discussion

Experiment 1 failed to replicate previous qualitative results in that we did not see the reported high accuracy. Although the average step widths were high, some individual participants exhibited response curves that were not staircase-shaped (Fig. 4B), at least for subintervals (Fig. 4C, first 100 trials). A longer period increased accuracy but violations of staircase-shaped response curves (Fig. 4B) occurred in that condition too. It is thus not the case that nonstepwise updating is a mere artefact of struggling with the task.

## Experiment 2

The purpose of Experiment 2 was twofold. Firstly, it was another attempt to replicate the patterns from previous studies. Upon carefully rereading them, we realised that participants had been informed about the nonstationarity of the latent probability distribution.^10^ In Experiment 2, we tested the effect of the availability of this information.^11^ Secondly, we tested the effect of the amount of effort required from a participant to change their probability estimate since we realised that it was generally greater in experiments reporting stepwise patterns compared with those that did not (our pilot; McGuire et al., 2014; Nassar et al., 2010; Norton et al., 2019). To address the possibility that the results of Experiment 1 arose due to poor motivation, we now incentivised accuracy (as in Khaw et al., 2017). One possible outcome is that the pattern from previous studies (e.g., Fig. 1) obtains in all four conditions, indicating that Experiment 1 failed to replicate due to insufficient participant motivation (remedied by Experiment 2 incentivising accuracy). This would suggest that staircase-shaped response curves are a general feature of (diligent) estimation of a Bernoulli probability. Another possibility is that only the cell with the particular design choices made in previous studies (information about nonstationarity and higher effort updating) replicate previous results, but that they do not hold under slightly different set-ups.

### Method

#### Participants

Sixty-two participants (mean age = 24.7 years, *SD* = 6.3) were recruited from university campuses in Uppsala. Data from two participants were excluded since they chose to terminate early. Of the remaining sixty participants, 47 identified as women, 11 as men, and two as other. Participants were rewarded with gift vouchers for a major Swedish book shop chain (Akademibokhandeln) with a total value determined by the (rounded) root-mean-squared error of their estimates, ranging between US$11 and US$28.^12^ Two participants in the No info, High effort condition, six in Info, High effort, six in No info, Low effort, and five in Info, Low effort received course credits instead of gift cards.

#### Design

Since participants in the Long period condition of Experiment 1 performed better on the task than those in the Short period condition, we chose to go with the Long period (500 trials) in Experiment 2. The experiment was a 2 × 2 factorial design where we varied (i) whether participants were told that the generative function was nonstationary and (ii) the response effort of updating the response slider. For (i), participants were either only given the written instructions from Gallistel et al. (2014) and Ricci & Gallistel (2017), which did not comment on stationarity (No information), or told that the contents of the box *might change after each draw*, that these changes would occur *throughout the task,* that the changes could be *fast or slow* and that their task was to track the proportion *as it changed* (Information). Effectively, they were told that the generative process was nonstationary. For (ii), one group of participants had the same response mode as in previous studies (Gallistel et al., 2014; Khaw et al., 2017; Ricci & Gallistel, 2017: High effort). To respond with the same estimate as last trial, they merely had to click the “Next” button (over which their cursor would already be hovering following their latest response), thus incurring minimal motor and time costs. To respond with a different estimate than last trial, they needed to move the mouse cursor to the slider, press the left mouse button and hold to drag the slider to the desired position, and then click the “Next” button. Providing a different estimate than on the last trial thus required greater response effort and took more time. For the other group, the cursor and “Next” button were removed and the slider was “stuck” to the mouse (Low effort): when the mouse was moved to the right, so was the slider and vice versa. Participants locked in their response and initiated the next trial by a mouse click. Providing the same estimate as last trial thus still required minimal motor and time costs but the costs of providing a different estimate were decreased.

#### Materials

The task was replicated from the code used in Ricci & Gallistel (2017; see General Methods).

#### Procedure

The procedure was as specified under General Methods.

### Results

We first consider the causal effects of providing the information that the distribution was nonstationary, decreasing the effort required to update the estimate, and their interaction, on our two summary statistics of interest, mean absolute error and mean step width. We then consider individual level data.

**Fig. 5:**
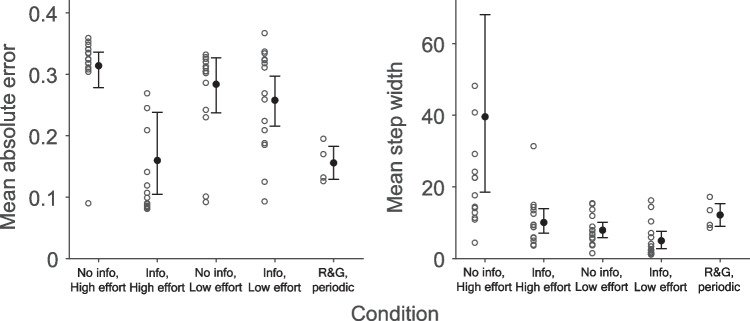
Results from Experiment 2. Mean absolute error and mean step width by condition and the periodic condition in Ricci & Gallistel (2017). Whiskers indicate 95% bootstrapped confidence intervals, circles indicate individual participants. One outlier in the Info, High effort condition and one outlier in the No info, Low effort condition with mean absolute errors of 0.615 and 0.466, respectively, are not displayed to preserve legibility. Two outliers in the No info, High effort condition with mean step widths of 126 and 195 are also off-scale

Being informed that the box might change decreased mean absolute error (*p* < 0.001). There was no effect of making it more or less effortful to update on mean absolute error (*p* = 0.19), but an interaction was found between information and effort (*p* = 0.015) such that accuracy is increased when participants are informed and it is more effortful to update—the condition most similar to previous studies (e.g., Ricci & Gallistel, 2017). The mean step width was decreased by being informed that the box might change (*p* < 0.001) and increased by making it more effortful to update (*p* < 0.001), especially when coupled with no prior information (*p* = 0.017). See Fig. 5. Thus, for both dependent measures the substantive effect lies in the interaction between response effort and information, where the joint effect of these variables influences the response curves. Note that only the cell with the particular design choices made in Ricci & Gallistel (2017) replicates their results. See Table 1 for descriptives.

**Table 1:** Descriptives for the dependent measures

**Mean absolute error**			
	No information	Information	Main effect
Low effort	0.284 (0.092)	0.258 (0.084)	0.271
High effort	0.314 (0.064)	0.160 (0.141)	0.237
Main effect	0.299	0.209	
**Mean step width**			
	No information	Information	Main effect
Low effort	7.922 (4.358)	4.979 (4.957)	6.450
High effort	39.571 (51.941)	10.076 (7.061)	24.823
Main effect	23.747	7.527	

Descriptives are presented as “mean (standard deviation)”. Main effects are marginal means

**Fig. 6:**
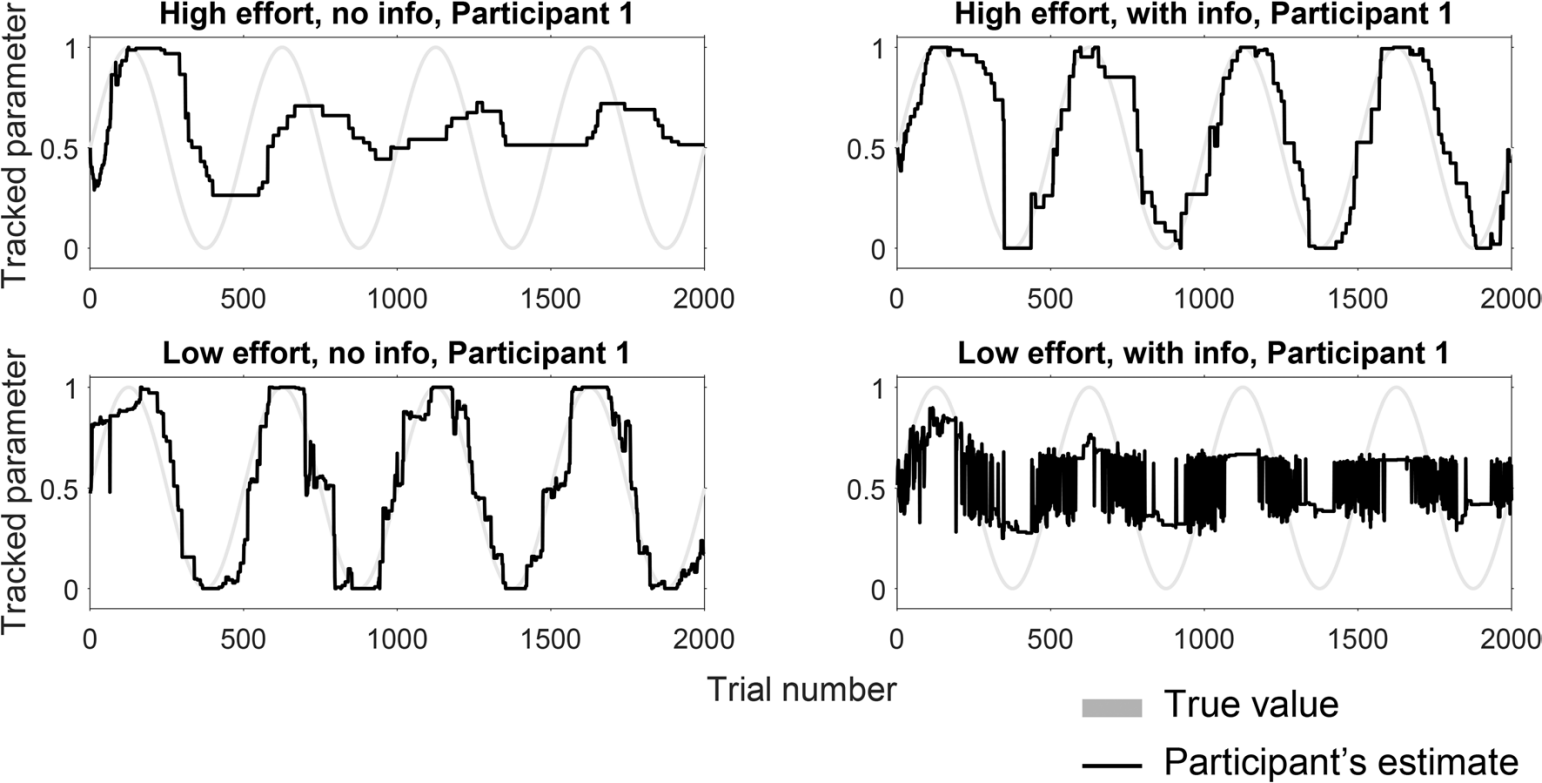
Example participants from Experiment 2. Provided probability estimate (x axis) by trial (y axis) for the first participant of each condition. Participant estimates in black, true probability in light grey

As is evident from Fig. 6, the characteristic combination of high accuracy and long step widths is not ubiquitous but obtains for some participants in certain subintervals of the data.

Also in Experiment 2, the main contributor to estimation error was other sources (between 71.1% and 81.8%). Lag accounted for between 13.3% and 20.2%. An insufficient number of updates accounted for less error in the Low effort conditions (No info, Low effort: 6.0%; Info, Low effort: and 4.9% vs. No info, High effort: 14.2%; Info, High effort: 12.9%). This could tentatively suggest that reducing the effort required to update invites a more appropriate number of adjustments. See the Supplement for details.

**Fig. 7:**
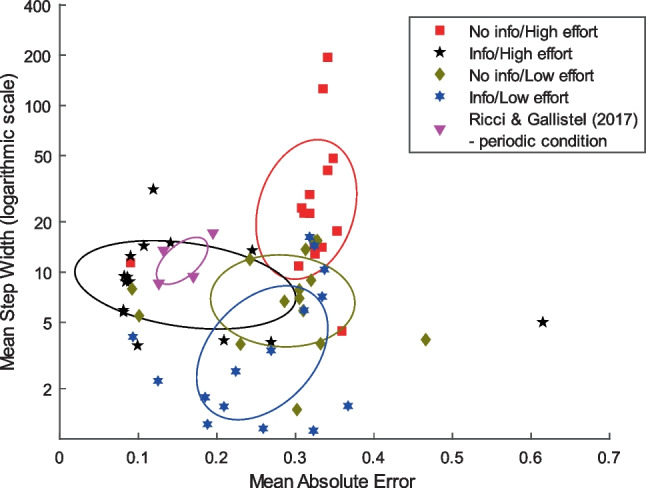
Summary statistics for individual participants. Mean step width and mean absolute error (MAE) for each participant in Experiment 2 and the periodic condition in Ricci & Gallistel (2017). Ellipses indicate one standard deviation from mean of fitted two-dimensional normal distributions. Although there are outliers, each condition tends towards a discernible cluster. The Info/High effort condition participants typically cluster close to participants from the periodic condition in Ricci & Gallistel (2017). Info/Low effort participants form a cluster of lower step widths, No info/High effort a cluster with higher step widths and No info/Low effort in betwen. Note the outlier with an MAE greater than 0.6. From visual inspection of their responses, we suspect they mixed up which end of the slider indicated “100% only blue”. Inverting their responses brings their MAE down to 0.385 and decreases all *p* values in the ANOVA but does not change any qualitative conclusions, see Supplement

A scatterplot of summary statistics for each individual participant (Fig. 7) shows that only the Info/High effort condition clusters around the data from Ricci & Gallistel (2017). The other conditions form separate clusters elsewhere. A number of highly accurate participants display low mean step widths, indicating that stepwise updating is not ubiquitous even for participants who are clearly on-task.

After completing the experiment task, participants were asked to draw a graph of the probability of drawing a blue ring over the course of the experiment. These data are not clear-cut and should be interpreted tentatively (see https://osf.io/zhv2r/ for all graphs) but one could perhaps divide them into three groups: some participants did not understand what to draw (e.g., Subject ID 3), some drew qualitatively incorrect graphs (e.g., linear, Subject ID 13), but some (about 35 out of 60) did manage to extract the back-and-forth pattern in the generative function.^13^ They struggled, however, with extracting the amplitude and period.

### Discussion

In Experiment 2, we found that design choices regarding prior information and updating effort affected the degree to which we obtained accurate, stepwise updating. Indeed, we only replicate Ricci & Gallistel’s (2017) results in the cell most similar to their design (Info, High effort).

The interaction effect of Information and Effort on mean absolute error and step width may suggest (to us) that participants actively infer what behaviour is reasonable from the task design. If they are told that the distribution is nonstationary and it is relatively effortful to change the estimate, they infer that the experimenter has set the generative function to change somewhat slowly, thus helping them to track it accurately. If they are not told anything about the distribution but it requires more effort to change the estimate, they infer that the experimenter chose to make updating effortful because they only need to do it occasionally. If this kind of “teleological” reasoning indeed is the explanation for the interaction, it underscores how important participants’ declarative beliefs are for their trial-by-trial responses.

## General discussion

These results show that the accuracy and step widths of response curves are not robust but sensitive to (fairly minor) design choices. We take them to suggest that people possess, at least, three capabilities. Firstly, people are able to estimate a nonstationary Bernoulli probability online—albeit with varying accuracy (Figs. 4 and 6). Secondly, they can modulate the estimation based on beliefs about the generative process (interaction effect on mean absolute error, Fig. 5). Thirdly, there seems to exist some kind of process which regulates when and how often the overt estimate is updated to correspond to the covert, internal estimate (see “response threshold”; Forsgren et al., 2023). This too appears modulated by declarative beliefs (interaction effect on mean step width). In the Supplement, we show that there are main effects of Effort on the mean size of updates when made (“step heights”) and the number of times the updating direction is reversed (“direction reversals”). Those results are consistent with an “economising” threshold that implements an accuracy–cost trade-off. In addition, and less surely, we found that people seem able to extract a vague, qualitative belief about the generative function which is retained at least for a while after they have completed the task (posttest drawings of generative function).

This invites the following speculation: maybe trial-by-trial updating is indeed how people *track* probabilities from moment to moment, but these estimates are never encoded in long-term memory. Instead, a belief about the generative function is extracted and stored (cf. Brehmer, 1980; Mcdaniel & Busemeyer, 2005). Online tracking thus affects beliefs but the reverse holds too: There exists a literature which investigates how learning rates can be *learnt* from experience (Behrens et al., 2007; Rushworth & Behrens, 2008) by estimating the volatility in the environment (Silvetti et al., 2013) but our results suggest that the learning rate can also be *calibrated* by beliefs.^14^ That is, it is not a question of either associative learning or hypothesis testing but always both and there exists a feedback loop between the two. If so, any theory that purely focuses on explaining the online estimates in the present paradigm, rather than encoded beliefs, would only give us insight into a very local form of probability learning. This includes various versions of the delta rule (e.g., Forsgren et al., 2023; Nassar et al., 2010).

Previous models in the Bayesian tradition (e.g., Mathys et al., 2014; Nassar et al., 2010; Piray & Daw, 2020) assume a (fixed) structural representation of the task but (flexibly) update that representation’s parameters. They thus allow effects of beliefs on online estimation by including priors that could be set by initial beliefs. Those priors will, however, eventually be overcome by data. This is not compatible with how some participants appear to maintain the qualitative characteristics of their response curves throughout the sessions (Fig. 4B, & Fig. 6; see also https://osf.io/zhv2r/). One way of explaining persistent effects of information would be through a model that, in parallel with online estimation, holds and (flexibly) tests hypotheses about the structural representation of the task (cf. Szollosi et al., 2023). In a nutshell, we envision a theory where people simultaneously possess the intuitive statistician’s capacity for data compression (Peterson & Beach, 1967) and the intuitive scientist’s capacity for structural modelling (Szollosi & Newell, 2020).

Future research should test the influences of experience versus beliefs on learning rates by varying prior information within-person and measuring some statistic related to information-discounting over the course of the task. It should also be investigated whether the response curves per se are encoded into long-term memory or whether the coding only preserves the gist of the generative function through posttests (cf. Mason et al., 2022). This is important since it may be our long-term memories that we act on when making real-world decisions.

Compared with any real-life probability estimation task the information about the generative function provided in the Information conditions here was extremely impoverished. When estimating the risk of the train coming in late, for example, we have a rich cognitive causal model (Sloman, 2005) of train punctuality and can use prior information (e.g., reading in the paper that there will be maintenance work done) to inform how we weight past observations or infer regime changes. A complete theory must account for how beliefs about such things can affect my estimate of the risk of the train being late but also how seeing other trains repeatedly come in on time can make me reevaluate my belief about the effects of the maintenance work.

## Supplementary Information

Below is the link to the electronic supplementary material.

Supplementary file1 (DOCX 410 kb)

## Data Availability

All data and materials are available at https://osf.io/zhv2r/.

## Footnotes

1 In Forsgren et al. (2023), we showed that the second (theoretical) part of that claim was premature: In a quantitative model comparison, previously published data were better explained by a combination of associative learning and sequential evidence accumulation than the hypothesis testing model. Here, we focus on scrutinising the first part of that claim regarding the empirical phenomenon.

2 An alternative dependent measure is the probability of making an update to the estimate (sometimes called “perseveration probability”, e.g. Nassar et al., 2021). This is a non-linear rescaling of mean step width. See the Supplement for plots and analyses of this additional measure.

3 Previous experiments provided similar information verbally to participants, but we had failed to notice this when designing Experiment 1.

4 We had access to their code and could thus verify that the two were equivalent.

5 Previous versions use red and green rings. We used red and blue rings to avoid issues with red–green colour blindness.

6 Although we measured fewer trials than previous experiments (Gallistel et al., 2014; Khaw et al., 2017; Ricci & Gallistel, 2017), those experiments have not reported any qualitative changes in behaviour between earlier and later trials.

7 All questionnaire data are available at https://osf.io/zhv2r/.

8 The four previous studies using the Bernoulli parameter paradigm have recruited 39 participants in total.

9 Calculated using 2017 OECD purchasing power parity estimates.

10 Robinson (1964): “The subjects were instructed in detail on the concept of a time-varying probability” (p. 10). Gallistel et al. (2014): “Subjects are told that the box of RINGS may sometimes be silently replaced by a box with a different proportion of green circles” (p. 101) Ricci and Gallistel (2017): “Subjects were told that the hidden distribution contained some proportion of green and red rings and that this proportion silently changes” (p. 1482). Khaw et al. (2017): “The subjects are not told when a change in the box occurs, but they are told in advance that the box might change, and they know the probability of a regime change and the distribution from which the new box is drawn” (p. 90).

11 We were unable to obtain the manuscript for the verbal information through private communication. We could therefore not exactly replicate the information provided, but it is unequivocal that participants in all studies were *at least* aware of the nonstationarity. We thus consider this a minimal treatment which may not include all information conveyed to participants in previous studies.

12 Calculated using 2017 OECD purchasing power parity estimates.

13 Ricci & Gallistel (2017) reported something similar: “During debriefing, all 4 subjects spontaneously remarked that the probability changed periodically” (p. 1488).

14 In the supplement, we show that there are main effects of Information on mean step heights and the number of direction reversals and argue that those results are consistent with this “calibration hypothesis”.

## Code availability

All code used to analyse the data is available at https://osf.io/zhv2r/.

## Declarations

### Open practices statement

All data, materials, and analysis code are available at https://osf.io/zhv2r/. This study was not preregistered.
