# Supplementary material for: Further perceptions of probability: Accurate, stepwise updating is contingent on prior information about the task and the response mode
Source: Psychon Bull Rev. 2024 Nov 14;32(3):1284–96. doi: 10.3758/s13423-024-02604-2 (PMC12092534; doi:10.3758/s13423-024-02604-2)
Supplement: Supplementary file 1 — Supplementary file1 (DOCX 410 kb) [file 13423_2024_2604_MOESM1_ESM.docx]

**Supplementary materials for “Further Perceptions of Probability: Accurate, Stepwise Updating is Contingent on Prior Information about the Task and the Response Mode”**

Mattias Forsgren, Peter Juslin and Ronald van den Berg

**Plot and permutation ANOVA for Experiment 2 without the outlier**

Inspecting the raw data for the outlier participant shows that they are for certain subintervals highly myopic but stabilise when the true probability is close to 0 or 1 (during the peaks and troughs of the sine curve; Figure A1). However, these stable intervals appear inverted: when the true probability is close to 1 the participant’s estimate is close to 0, and vice versa. We therefore suspect that they mistook which end of the response scale indicated “100% only blue” and which indicated “0% no blue”.

We invert the mean absolute error (MAE) for the outlier participant to obtain an MAE of 0.385. Rerunning the ANOVA with these data gives us p-values for the effects of Info, Effort, and their interaction of 4.60⸱10^–5^, 0.059, and 1.80⸱10^–3^. Thus, although the p-values are clearly lower, they do not give any reason to change the conclusions drawn in the main body.

We also redraw Figure 7 after having inverted the outlier participant’s MAE and find that this narrows the ellipse for their condition by about a third (Figure A2).

**ANOVAs on probability of updating (“perseveration probability”)**

A reviewer suggested that we include the probability of making an update to the estimate (sometimes called “perseveration probability”, e.g. Nassar et al., 2021) as an alternative to mean step width. The two measures have the following relationship:

$$\begin{aligned} Perseveration probability= 1-\frac{N. of updates}{Total N. of trials}\#(1) \end{aligned}$$

$$\begin{aligned} \frac{Total N. of trials}{N. of updates}=Mean step width\#(2) \end{aligned}$$

With minimal algebra we obtain that:

$$\begin{aligned} Perseveration probability=1-\frac{1}{Mean step width}\#(3) \end{aligned}$$

Thus, perseveration probability is a non-linear rescaling of mean step width according to a hyperbola. From the descriptive plots of this new measure (Figure B1) it is evident that the difference between No info, High effort and the other conditions is decreased due to the hyperbolic rescaling. Moreover, a rerun of the permutation ANOVAs shows that under this new measure, there is still no evidence of an effect of Period length in Experiment 1 (p = 0.379). In Experiment 2, the main effect of Info is unchanged (p < 0.001) but what previously was an interaction effect (p = 0.082) is now mainly attributable to a main effect of Effort (p < 0.001). Our take on this is that the rescaling conceals the substantial differences in behaviour between No info, High effort and the other conditions that are observable in the raw response data.

**Error decomposition**

One of the main findings reported in the main body is that experimental manipulation of the instructions and response mode affected both the accuracy with which participants tracked the latent probability and the frequency with which they updated their estimates. Importantly, these two variables are not fully independent, as the maximum accuracy for tracking the hidden probability is constrained by the frequency of slider updates. Fewer updates makes the response vector more discrete, limiting its ability to precisely follow the smooth, sinusoidal pattern of the to-be-tracked probability. To investigate the relation between these variables in more detail, we decompose the error of each participant into three components. Firstly, errors could arise because participants make too few updates of their estimates (“Discreteness”), as explained above. If this accounts for a large proportion of the total error then it would suggest that participants persevere with their estimates for too long. We calculate this proportion by comparing each participant’s error to the minimal error attainable if one makes the same number of updates as the participant. That is, as $\frac{\mathrm{RMSE}_{\mathrm{minimal}}}{\mathrm{RMSE}_{\mathrm{empirical}}}$ where $\mathrm{RMSE}_{\mathrm{minimal}}$ is the minimal attainable root mean squared error if one makes the same number of updates as the participant and $\mathrm{RMSE}_{\mathrm{empirical}}$ is the actual root mean squared error of the participant. The former is difficult to compute exactly, but can be approximated using numerical methods (Bergerhoff et al., 2019). Secondly, errors could arise because the participant’s estimate lags behind the true parameter value (“Lag”). If this lag would account for a large proportion of the total error then it would suggest that participants struggle with keeping up with the change in the parameter value. We calculate this proportion by comparing each participant’s error to the minimal error attainable by any backwards shift of the participant’s estimates. Code for these calculations is provided at https://osf.io/zhv2r/.

The results (Table C1) show that these two explanations account for relatively small proportions of the total error of participants. The fact that Discreteness accounts for a larger proportion of the errors in the Short period condition, Experiment 1, suggests to us that participants do not adapt the number of updates they make to the change rate of the latent probability sufficiently. That Discreteness accounts for a substantially smaller proportion of the error for the Low effort conditions, Experiment 2, suggests to us that reducing the effort required to update does indeed facilitate making an appropriate number of adjustments.

**Table C1.** Mean percentage of error explained by insufficient number of updates (‘Discreteness’), lagging estimates, and other explanations, by experiment and condition.

|  | **Discreteness** | **Lag** | **Other** |
| --- | --- | --- | --- |
| **Experiment 1** |  |  |  |
| Short period | 32.5% | 10.5% | 57% |
| Long period | 12.4% | 14.4% | 73.2% |
| **Experiment 2** | | | |
| No info, High effort | 14.2% | 14.8% | 71.1% |
| Info, High effort | 12.9% | 20.2% | 66.9% |
| No info, Low effort | 6% | 13.8% | 80.1% |
| Info, Low effort | 4.9% | 13.3% | 81.8% |

**Effects of the experimental manipulations on other response curve characteristics**

In the main analyses we examined how the experimental manipulations affect the step width and accuracy of participants’ response curves. Those two characteristics were chosen based on their prominence in previous literature. Here, we investigate two additional summary statistics to better understand how the response curves differ between conditions (on average) and through what mechanisms the manipulations could affect accuracy. The first measure is the size of the changes in the estimate when an update is made (“step height”, Gallistel et al., 2014). The other is the number of times that an update is made in the opposite direction of the previous estimate (“direction reversals”). A high value on this metric implies that the graph of that participant’s estimates looks “fuzzy”, jittering back and forth, while a low value implies a smoother tracking of the Bernoulli parameter.

The results (Figure C1) show clear main effects in Experiment 2 which are confirmed by permutation ANOVAs. Firstly, information that the Bernoulli parameter is non-stationary makes participants make larger changes to their estimates when they update it (p < 0.002). Secondly, decreasing the effort needed to update also decreases the mean step height (p < 0.001) Thirdly, participants reverse the direction of their updates particularly often when informed of the non-stationarity (p < 0.001). Lastly, decreasing the effort required to update increases the number of direction reversals (p < 0.001). There is no appreciable evidence of other effects (p ≥ 0.23).

In connection with the main effects on step widths reported in the main body, these main effects of effort suggest to us that participants employ some kind of “economising” adaptive response threshold that implements an accuracy-cost trade off. When it is “costly”, in terms of effort (and time) to update, fine tuning of estimates is decreased (decreasing direction reversals) but step heights are increased to keep up with the changing Bernoulli parameter. The main effects of information, on the other hand, are interpreted by us as the (covert) learning rate being calibrated by beliefs. When prior information makes participants believe (or believe with greater certainty) that the generative function is changing, they increase their learning rate. For a given step width, this leads to greater step heights. A higher learning rate also naturally leads to “fuzzier” response curves. Spurious sequences in the sampling will “pull” the estimate further, increasing the likelihood of it surpassing the response threshold. The lack of effects of period length on mean step height (p = 0.29), direction reversals (p = 0.57), and mean step widths (see main body) could be interpreted as the period not affecting the estimation process per se, only the performance by determining the difficulty of the task.

**References**

Bergerhoff, L., Weickert, J., & Dar, Y. (2019). Algorithms for Piecewise Constant Signal Approximations. *2019 27th European Signal Processing Conference (EUSIPCO)*, 1–5. https://doi.org/10.23919/EUSIPCO.2019.8902559

Gallistel, C. R., Krishan, M., Liu, Y., Miller, R., & Latham, P. E. (2014). The perception of probability. *Psychological Review*. https://doi.org/10.1037/a0035232

Nassar, M. R., Waltz, J. A., Albrecht, M. A., Gold, J. M., & Frank, M. J. (2021). All or nothing belief updating in patients with schizophrenia reduces precision and flexibility of beliefs. *Brain*, *144*(3), 1013–1029. https://doi.org/10.1093/brain/awaa453
